# Dose and life stage-dependent effects of dietary beta-carotene supplementation on the growth and development of the Booroolong frog

**DOI:** 10.1093/conphys/coy052

**Published:** 2018-09-19

**Authors:** Leesa M Keogh, Aimee J Silla, Michael S McFadden, Phillip G Byrne

**Affiliations:** 1 School of Earth, Atmospheric and Life Sciences, University of Wollongong, NSW, Australia; 2 Herpetofauna Department, Taronga Conservation Society Australia, Mosman, NSW, Australia

**Keywords:** Amphibian, antioxidant, captive breeding, nutrition, tadpole

## Abstract

Carotenoids are known for their antioxidant capacity and are considered to play an important role in vertebrate growth and development. However, evidence for their beneficial effects remains limited, possibly because very few studies have tested for dose effects across different life stages. The present study investigated the effect of various doses of dietary beta-carotene supplements on the growth and development of larval and post-metamorphic Booroolong frogs (*Litoria booroolongensis*). Larval and post-metamorphic basal diets (containing 0.015 and 0.005 mg g^−1^ total carotenoids, respectively) were supplemented with beta-carotene at one of four concentrations: 0 mg g^−1^, 0.1 mg g^−1^, 1 mg g^−1^ and 10 mg g^−1^. Each treatment included 72 replicate individuals, and individuals remained on the same diet treatment over both life stages (spanning 53 experimental weeks). Our results show that larvae receiving an intermediate (1 mg g^−1^) beta-carotene supplement dose grew faster than unsupplemented larvae (0 mg g^−^^1^), and metamorphosed earlier. After metamorphosis, there was no effect of the lowest supplement dose (0.1 mg g^−1^) on growth and development. However, juveniles fed the highest supplement dose (10 mg g^−1^) displayed significantly smaller body mass and lower body condition, compared to all other supplement doses, from 4-months through to sexual maturity (7-months). These findings indicate that beta-carotene supplementation has positive effects on growth and development, but only at intermediate doses, and only in the larval life stage. This knowledge may assist with amphibian conservation by expediting the rate that metamorphs can be generated in captive breeding programmes. More broadly, this is the first study to demonstrate both dose and life stage-dependent effects of dietary beta-carotene supplementation on vertebrate growth and development.

## Teaser

This study is the first to show that dietary beta-carotene supplementation has a dose and life stage-dependent effect on vertebrate growth and development. Knowledge in this domain could assist with the captive breeding of threatened species.

## Introduction

Dietary carotenoids are expected to directly impact vertebrate growth and development by acting as powerful antioxidants. Growth is associated with high metabolic activity and oxygen consumption, accompanied by free radical production and potential for oxidative stress (Ogilvy *et al.*, 2012). By scavenging and quenching free radicals produced during metabolism, carotenoids can reduce the oxidative damage caused to cells, tissues and DNA, enabling more efficient cell division and differentiation (Edge *et al.*, 1997), and consequently increased growth (Ogilvy and Preziosi, 2012). In turn, an increase in growth (and more rapid changes in body size) should facilitate more rapid progression through developmental stages (Gillooly *et al.*, 2002; Marri and Richner, 2014). Additionally, dietary carotenoids might influence growth and development indirectly by improving immune function and general health (Ogilvy and Preziosi, 2012), or foraging performance (Toomey and McGraw, 2011).

Despite sound theoretical arguments suggesting that carotenoid supplementation will improve vertebrate growth and development, this remains strongly debated because evidence for beneficial effects remains equivocal, both within and between taxa. In some species, clear benefits of carotenoid supplementation have been reported (e.g. Torrissen, 1984; Christiansen and Torrissen, 1996; Cucco *et al.*, 2006; Ogilvy and Preziosi, 2012; Arulvasu *et al.*, 2013). However, in other species there have been no detectable benefits (e.g. Řehulka, 2000; Amar *et al.*, 2004; Wang *et al.*, 2006; Byrne and Silla, 2017), or negative effects (Cothran *et al.*, 2015). This interspecific variation might exist because species differ in their capacity to utilize carotenoids, reflecting different evolutionary histories in carotenoid consumption. For instance, because herbivores consume higher amounts of carotenoids than carnivores, they might have a greater physiological capacity to absorb and process these micronutrients (Tella *et al.*, 2004). Alternatively, much of the interspecific variation reported in the literature might simply reflect the fact that very few studies have tested for dose–response relationships.

Within species, the effect of dietary carotenoids on growth and development may shift depending on the quantities of carotenoids consumed. Based on optimization theory, we should expect that benefits will be restricted to a specific range of metabolically-relevant concentrations, with growth and development optimized at intermediate concentrations (Christiansen *et al.*, 1995; Christiansen and Torrissen, 1996). If carotenoids are provided at extremely low concentrations, effects on growth and development might be negligible, and any benefits might remain undetected. In contrast, carotenoids at extremely high concentrations may stunt growth and development because high concentrations could be toxic, and large amounts of energy might be required to detoxify and remove excess nutrients, limiting growth (Palozza *et al.*, 1997; Cothran *et al.*, 2015). High concentrations could also have pro-oxidant effects, whereby excess carotenoids elicit an increase in oxidative stress, either directly by generating additional ROS (Stoss and Refstie, 1983), or indirectly by suppressing the endogenous antioxidant system (Bouayed and Bohn, 2010). Adding to this complexity, is the possibility that optimal carotenoid concentrations differ between life stages. For most vertebrate species growth will be most rapid during juvenile life stages, so we should expect that benefits associated with ROS scavenging will be most pronounced during early development. Surprisingly, however, past studies testing for effects of carotenoids on growth have largely ignored the potential for life-stage effects, instead focussing on very narrow periods of either juvenile or adult development.

Anuran amphibians (frogs and toads) provide a model group for investigating carotenoid dose–response relationships across different life stages because most species have a biphasic life cycle, with juvenile and adult life stages separated by a distinct period of metamorphosis (Wilbur and Collins, 1973). At each life stage, anurans consume different types of food (in general larvae eat algae and adults eat invertebrates), creating life stage specific differences in carotenoid intake. Growth is usually several orders of magnitude faster during the larval life stage, and assuming that this is when ROS production is highest, this is the life stage when the antioxidant properties of carotenoids will be most beneficial. Antioxidant properties may also be valuable during and immediately after metamorphosis. At this developmental phase, rapid cell division and morphological change can dramatically increase ROS production (Blount *et al.*, 2000). Moreover, a pronounced spike in cellular oxygen concentration during the transition from aquatic to terrestrial life would be expected to cause oxidative stress (Ogilvy *et al.*, 2012). To date, the effect of dietary carotenoids on growth and development has been examined in six anuran species, with a focus on the combined effects of five common carotenoids; astaxanthin, cryptoxanthin beta-carotene, lutein and zeaxanthin (Ogilvy and Preziosi, 2012; Ogilvy *et al.*, 2012; Dugas *et al.*, 2013; Cothran *et al.*, 2015; Byrne and Silla, 2017). As for studies in other vertebrates, studies in amphibians have mainly tested for effects at one dose or life stage, and findings have been inconsistent. Despite these inconsistencies, benefits to growth and development have been reported for a number of anuran species from several families (Ogilvy and Preziosi, 2012; Ogilvy *et al.*, 2012; Dugas *et al.*, 2013), suggesting that carotenoid-mediated growth benefits could be widespread in amphibians.

From an applied perspective, knowledge of carotenoid dose effects on amphibian growth and development may have considerable conservation value. Globally, amphibians are declining faster than any other vertebrate class and the recommended conservation action for threatened species is captive breeding and reintroduction (Gascon *et al.*, 2007). To date, however, the success of captive breeding programs has been limited, in part due to a lack of knowledge regarding amphibian nutritional requirements. Identifying whether specific doses of carotenoids improve anuran growth and development, and whether the importance of carotenoids varies between life stages, may help recovery teams more effectively generate mature individuals needed to maintain viable captive insurance colonies. Furthermore, rapid generation of healthy animals could increase the number of individuals available for release, which is known to be an important predictor of reintroduction success (Tarszisz *et al.*, 2014).

The present study aimed to investigate the influence of beta-carotene on the growth and development of the critically endangered Booroolong frog *Litoria booroolongensis*. The specific aim was to determine whether effects of dietary beta-carotene supplementation on growth and development are either dose (0, 0.1, 1 and 10 mg g^−1^) or life-stage (larval and post-metamorphic) dependent.

## Methods

The procedures outlined below were performed following approval by the University of Wollongong’s Animal Ethics Committee (approval number AE 14/21).

### Study species

*Litoria booroolongensis* is listed by the IUCN as critically endangered (Hero *et al.*, 2004) and in 2008, under the recommendation of the NSW Department of Environment and Heritage (OEH), a captive breeding program for the species was established at Taronga Zoo (Sydney, Australia).

### Experimental animals

A total of 360 *L. booroolongensis* eggs (from four discrete clutches) were collected from a captive colony maintained at Taronga Zoo. Egg clutches were laid between 02 January 2015 and 17 January 2015, and all eggs hatched within 3 days of oviposition. Within 1–2 days of being laid, egg clutches were transported to the Ecological Research Centre at the University of Wollongong. Individual clutches were held in ~700–1000 ml of carbon-filtered water in plastic aquarium bags enriched with atmospheric oxygen. Hatchling tadpoles from each clutch were held communally in 20-l aquaria (one clutch per aquaria) for a 2-week period prior to being transferred to individual experimental containers. Immediately after hatching, tadpoles were fed a basal diet of ground fish flake mixture (75:25 Sera Flora/Sera Sans; SERA, Heinsberg, Germany), *ad libitum*, every 2 days before the beginning of the experiment. A 2-week acclimation period was imposed in order to ensure all tadpoles were feeding properly and were of similar size at the beginning of the experiment. All tadpoles were 9–15 days old post-hatching at the commencement of the experiment.

### Experimental design

Individuals were reared on one of four diets supplemented with different concentrations of beta-carotene. Beta-carotene was selected because it is one of the major carotenoids found in algae and herbivorous insects consumed by anurans (Takaichi, 2011), has been shown to significantly impact anuran reproduction (Ogilvy *et al.*, 2012), and has been identified in the skin tissue of wild Booroolong frogs (B. Tinning, 2016, pers. comm.). Larval and post-metamorphic basal diets (containing 0.015 and 0.005 mg g^−1^ total carotenoids, respectively) were supplemented with beta-carotene at the following concentrations: 0 mg g^−1^ (control), 0.1 mg g^−1^ (low), 1 mg g^−1^ (medium) and 10 mg g^−1^ (high). These supplement doses were chosen because they encompassed, and expanded on those previously used to investigate the effects of dietary carotenoids on amphibian growth, and other fitness-determining traits (Ogilvy *et al.*, 2012; Dugas *et al.*, 2013; Silla *et al.*, 2016). Each treatment included 72 replicate individuals (288 individuals in total) and individuals remained on the same diet treatment throughout both larval and post-metamorphic life stages. The experiment commenced on 19 January 2015 and the larval stage of the experiment was completed by 13 July 2015, when the last tadpole metamorphosed. The post-metamorphic stage of the experiment commenced when the first individual metamorphosed on 3 March 2015 and was completed by 26 January 2016, when the last frog reached 7-months of age post-metamorphosis, and sex could be determined.

Animals (*n* = 288) were individually housed in plastic containers (10 cm diameter and 10.5 cm high) throughout both life stages. Each experimental container was enclosed in a black plastic ring so that there was no visual contact between individuals. The experimental containers were held in sets of nine in plastic trays, which were positioned across three shelves in a constant temperature room. The experimental containers were aligned in rows of three, with each diet treatment alternating between rows in the following order; 0, 0.1, 1 and 10 mg g^−1^ (see Supplementary Fig. S1). The room was artificially illuminated on a 15:9-h, day: night cycle (including twilight lighting for half an hour each cycle to simulate dawn and dusk). In addition to overhead room lighting, UV-B lights (Reptisun 10.0 UV-B 3600 bulb; Pet Pacific, Emu Plains, Australia) were suspended ~20 cm above each container, providing 6.5 h per day of UV-B light (between 10 am to 4:30 pm). Ambient temperature in the room was maintained at 22°C (range was 21.9–23°C).

### Larval husbandry and nutrition

During the larval life stage, containers were filled with 550 ml of carbon-filtered water. Water changes and water quality testing were conducted according to methods described previously (Keogh *et al.*, 2018). Over the duration of the experiment, ammonia concentrations remained low (<0.50 mg l^−1^), nitrate remained at 0 mg l^−1^ and pH remained at 7.4. Tadpoles were fed one of the four diets supplemented with different concentrations of beta-carotene (0, 0.1, 1 and 10 mg g^−1^; Supplementary Table S1). Each diet consisted of 990 mg of ground fish flake mixture and a combination of beta-carotene (0.1, 1 and 10 mg g^−1^) and cellulose microcrystalline powder (435 236; Sigma-Aldrich, Castle Hill, NSW; Supplementary Table S1). Total carotenoid concentrations in the basal diet were <0.015 mg g^−1^ (see Suppementary Table S2 for a carotenoid profile). Cellulose was used to ensure that each experimental diet consisted of the exact same quantity of feed. Cellulose is commonly used to create balanced designs in dietary studies because it has no nutritional value, is odourless, tasteless and cannot be digested (Dias *et al.*, 1998; Deng *et al.*, 2006). Importantly, in a dietary study run in parallel to our main experiment, cellulose was found to have no effect on the survivorship, growth or development of *L. booroolongensis* larvae (see Supplementary Table S3). Experimental diets were pre-prepared by suspending 1000 mg of feed in 10 ml of reverse osmosis water (RO water; Sartorius Stedim Biotech, Goettingen, Germany). Feed suspensions were stored in 20 ml syringes and frozen in opaque containers at −20°C until required. On feeding days, syringes were defrosted at room temperature, homogenised and administered in a drop-wise manner to ensure an even proportion of feed was administered to each individual. Tadpoles were fed two drops of feed suspension (dry mass; 0.0184–0.0291 g) for the first 4-weeks of the experiment, and then three-drops (dry mass; 0.0415–0.0706 g) for the remainder of the experiment (to support increased energetic demands). Tadpoles were fed treatment diets bi-weekly (Monday and Friday) for the duration of the experiment. This feeding regime ensured animals were fed *ad libitum*.

### Quantifying larval survival, growth and development

To quantify tadpole survivorship, each individual was observed every second day and scored as alive or dead. To quantify tadpole growth, each individual was photographed once per fortnight using a Panasonic Lumix DMC-FT20 camera. To ensure accurate photos, water volume in each experimental container was dropped to 100 ml before taking the photo, and then immediately refilled to 550 ml. The development of individual tadpoles was assessed every 2 days using Gosner staging tables. At the emergence of forelimbs (Gosner stages 43–46), the water volume in each experimental container was dropped to 100 ml. To facilitate metamorphosis, a piece of sponge (diameter 9 cm) and a thin layer (<1 cm) of aquarium stones was added to the bottom of each container, and placed on an angle to allow metamorphosing tadpoles to climb from the water. The tadpoles were not fed during this developmental period as tail reabsorption met their nutritional needs. Tadpoles were checked daily, and once an individual had completely reabsorbed its tail, it was blotted with absorbent tissue (kimwipes), weighed and photographed next to a scale. The day of complete tail reabsorption was used to quantify time to metamorphosis. Images of tadpoles and metamorphs were used to determine body length using image analysis software (ImageJ version 1.45s). For each tadpole, head and tail lengths were measured separately, and total tadpole length was calculated by summing these two measures. For each metamorph, snout-vent length was measured. For each individual, two measurements were made and values averaged.

### Post-metamorphic husbandry and nutrition

At metamorphic climax (Gosner stage 46), individuals were moved from their larval housing into new containers, which remained in original shelf positions. The post-metamorphic containers and cleaning regime used have been described previously (Keogh *et al.*, 2018). Post-metamorphosis, frogs were fed twice a week with 2–4 day old crickets (*Acheta domestica*) until they reached sexual maturity at 7 months of age. Total carotenoid concentrations in the basal diet were <0.006 mg g^−1^ (see Supplementary Table S2 for a carotenoid profile). Beta-carotene was supplemented by dusting the crickets with 0, 0.1, 1 or 10 mg g^−1^ of beta-carotene powder. Diets were again balanced using cellulose to control the overall quantity of feed that frogs received. In addition, to ensure healthy bone growth all individuals received calcium supplementation by adding 200 mg of calcium powder containing vitamin D3 (Repti-Cal, Aristopet, Melbourne, Australia) to each gram of treatment powder.

### Quantifying post-metamorphic survival, growth and development

To quantify adult survivorship, individuals were observed every second day and scored as alive or dead. To quantify adult growth rate, each individual was photographed and weighed every four weeks post-metamorphosis. Frogs were removed from their experimental containers, blotted dry with absorbent tissue paper and weighed to the nearest 0.01 g. Frogs were then photographed using a Panasonic Lumix DMC-FT20 digital camera. The snout-vent length [measured from the tip of the nose (snout) to the bottom of the anus (vent)] was measured for each individual from digital images using image analysis software (ImageJ, version 1.45s). For each individual, two measurements were made and values averaged. An index of body condition was calculated by taking the residuals of an ordinary least squares linear regression of body mass (g) against SVL (mm). Once the frogs reached sexual maturity (at 7 months post-metamorphosis), they were euthanised and sex determined following dissection.

### Statistical analyses

The effect of supplement dose on: (i) larval survival until metamorphosis (0–8 weeks post-hatching), and (ii) metamorph survival until sexual maturity (0–7 months post-metamorphosis) was tested using likelihood ratio chi-squared tests. Two tadpoles and six metamorphs were excluded from each analysis because they died as a result of unexpected adverse events. Linear mixed effects models fitted with restricted maximum likelihood (REML) were used to test the effect of supplement dose (0, 0.1, 1 and 10 mg g^−1^) on: (i) tadpole body length (measured from snout tip to tail tip) at experimental weeks 0, 2, 4, 6 and 8, (ii) the body mass (g) of individuals at metamorphosis, and (iii) the time taken (days) until metamorphosis. In each model the treatment dose was the fixed categorical effect and clutch number (1–4) was included as a random effect. Linear mixed effects models fitted with restricted maximum likelihood (REML) were also used to test the effect of treatment dose on metamorph mass, and metamorph body condition each month for seven months post-metamorphosis. In these models, supplement dose and individual sex were the fixed categorical effects, and clutch number (1–4) was a random effect. To normalize data and stabilize variances, we log transformed all response variables before running analyses. For LME models that detected significant effects, differences between treatment levels were compared using post hoc Tukey HSD tests. All statistical analyses were performed using JMP 11.0 software package (SAS Institute Inc. North Carolina, USE).

## Results

### Larval survival

Overall, 90.9% (260/286) of tadpoles survived to metamorphosis, with high survivorship in all supplement doses (0, 0.1, 1 and 10 mg g^−1^ beta-carotene; 90.3%, 93.1%, 90. % and 90.1% respectively). Differences in survival between treatment doses were not statistically significant (Chi^2^: *Χ*^2^ = 0.57, *df* = 3, *P* = 0.904).

### Larval growth

**Figure 1:**
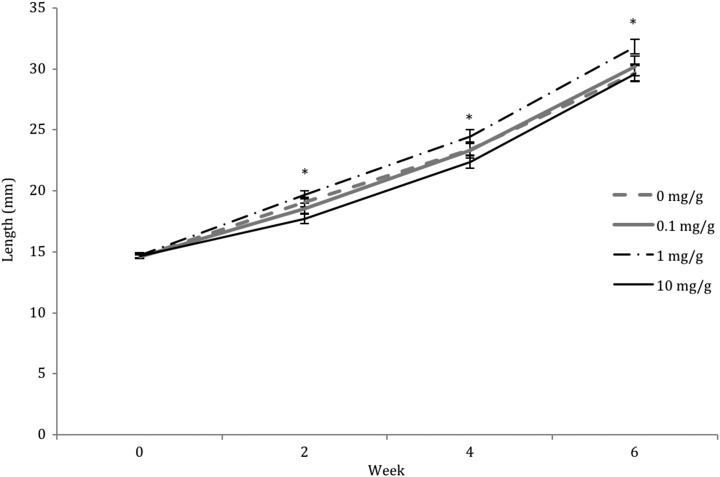
Effect of dietary beta-carotene supplementation (0, 0.1, 1 and 10 mg g^−1^) on tadpole body size (body and head length) over 8 experimental weeks in the booroolong frog (*n* = 260). Data presented are untransformed tadpole length means ± SEM. * denotes experimental weeks where treatments are significantly different (>0.05).

There was no significant effect of supplement dose on tadpole size (body length) at week 0 (LME: *F*_3,253_ = 0.33, *P* = 0.804; Fig. 1). However, in each subsequent sampling week (weeks 2, 4 and 6) supplement dose had a highly significant effect on tadpole size (LME: week 2: *F*_3,253.1_ = 5.49, *P* = 0.001; week 4: *F*_3,253_ = 3.13, *P* = 0.026; week 6: *F*_3,253_ = 3.50, *P* = 0.026). Tadpoles receiving a dose of 1 mg g^−1^ beta-carotene consistently showed the largest body size, while tadpoles receiving a dose of 10 mg g^−1^ consistently showed the smallest body size (see Fig. 1). At week 2, tadpoles supplemented with doses of 0 and 1 mg g^−1^ were significantly larger than those supplemented with a dose of 10 mg g^−1^ (Tukey’s HSD test: *P* = 0.047 and *P* = 0.001, respectively). At week 4, tadpoles supplemented with 1 mg g^−1^ beta-carotene were significantly larger than those supplemented with a dose of 10 mg g^−1^, (Tukey’s HSD test: *P* = 0.014), and at week 6, tadpoles supplemented with 1 mg g^−1^ were significantly larger than those supplemented with either 0 or 10 mg g^−1^ (Tukey’s HSD test: *P* = 0.044 and *P* = 0.024, respectively).

### Time to metamorphosis

**Figure 2:**
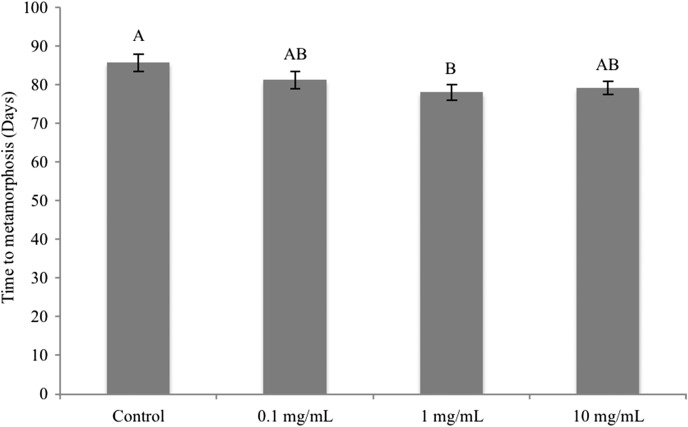
Effect of dietary beta-carotene supplementation (0, 0.1, 1 and 10 mg g^−1^) on time to metamorphosis in booroolong frogs (*n* = 260). Data presented are untransformed means ± SEM. Experimental treatments connected by the same letter are not significantly different from one another.

The time tadpoles took to metamorphose was highly variable, ranging from 49 to 175 days (mean ± SEM = 81.06 ± 2.042 days), and there was a significant difference in time to metamorphosis among diet treatments (LME: *F*_3,253_ = 3.85, *P* = 0.010). Tadpoles supplemented with a dose of 1 mg g^−1^ metamorphosed 1–8 days faster than all other supplement doses (0, 0.1 and 10 mg g^−1^), and the difference in time to metamorphosis between tadpoles supplemented with a dose of 0 mg g^−1^ and those supplemented with a dose of 1 mg g^−1^ was significant (Tukey’s HSD test: *P* = 0.006; Fig. 2).

### Body size at metamorphosis

The average body mass of metamorphs was 276.21 ± 5.92 mg (range: 190–160 mg). There was no significant difference among supplement doses (0, 0.1, 1 and 10 mg g^−1^) in body mass at metamorphosis (LME: body mass: *F*_3,253.1_ = 0.83, *P* = 0.478).

### Post-metamorphic survival

Overall, 83.9% (213/254) of individuals survived through to adulthood (sexual maturity). There was high survivorship in all diet treatments (0, 0.1, 1 and 10 mg g^−1^; 82.8%, 84.9%, 82.3% and 85.5%, respectively) and survivorship did not statistically differ between diet treatments (Chi^2^: *Χ*^2^ = 0.34, *df* = 3, *P* = 0.953).

### Post-metamorphic growth

**Figure 3:**
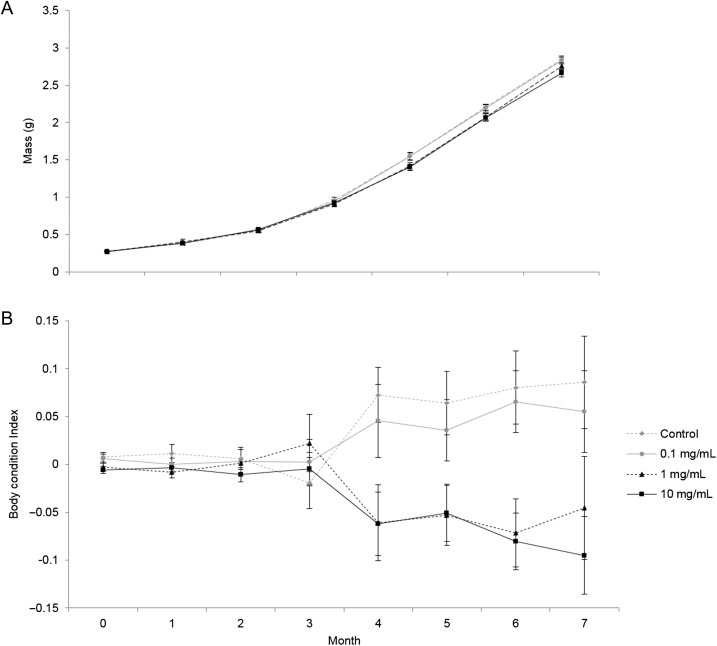
Effect of dietary beta-carotene supplementation (0, 0.1, 1 and 10 mg g^−1^) on (**A**) adult body mass (g) and (**B**) adult body condition over 7 experimental months in the booroolong frog (*n* = 213). Data presented are untransformed means ± standard error mean (SEM). * denotes experimental months where treatments are significantly different (>0.05).

There was no significant effect of supplement dose on adult body mass at months 0, 1, 2 or 3 (LME: month 0: *F*_3,205.2_ = 0.26, *P* = 0.853; month 1: *F*_3,205.6_ = 0.29, *P* = 0.832; month 2: *F*_3,205.1_ = 0.89, *P* = 0.447; month 3: *F*_3,205.1_ = 0.97, *P* = 0.409). However, at months 4, 5, 6 and 7 there was a significant effect of supplement dose on post-metamorphic body mass (LME: month 4: *F*_3,205.1_ = 4.09, *P* = 0.008; month 5: *F*_3,205.1_ = 3.08, *P* = 0.029; month 6: *F*_3,205.1_ = 3.34, *P* = 0.020; month 7: *F*_3,205.2.1_ = 2.64, *P* = 0.051). Frogs supplemented with 10 mg g^−1^ consistently exhibited a smaller body mass compared to frogs supplemented with all other doses (0, 0.1 and 1 mg g^−1^; Fig. 3a). At month 4, frogs supplemented with a dose of 1 mg g^−1^ were significantly smaller than those supplemented with 0 mg g^−1^ (Tukey’s HSD test: *P* = 0.039). At month 5, frogs supplemented with either 1 or 10 mg g^−1^ were significantly smaller than those supplemented with 0 mg g^−1^ (Tukey’s HSD test: *P* = 0.026 and *P* = 0.037 respectively), and at month 6, frogs receiving 10 mg g^−1^ were significantly smaller than those supplemented with 0 mg g^−1^ (Tukey’s HSD test: *P* = 0.025).

Sex did not have a significant effect on frog weight at months 0 to 6 (LME: month 0: *F*_1,207.7_ = 2.25, *P* = 0.135; month 1: *F*_1,204.7_ = 0.06, *P* = 0.816; month 2: *F*_1,207.5_ = 0.52, *P* = 0.473; month 3: *F*_1,206.7_ = 0.58, *P* = 0.447; month 4: *F*_3,208.8_ = 0.32, *P* = 0.5709; month 5: *F*_1,207_ = 0.00, *P* = 0.997; month 6: *F*_1,207.5_ = 0.02, *P* = 0.880). However, at month 7 there was a significant effect of sex on frog weight (LME: *F*_1,207.8_ = 6.26, *P* = 0.0131), with females being significantly heavier than males.

### Post-metamorphic body condition

There was no significant effect of supplement dose on adult body condition at months 0, 1, 2 or 3 (LME: month 0: *F*_3,205.2_ = 1.22, *P* = 0.304; month 1: *F*_3,205.2_ = 1.48, *P* = 0.220; month 2: *F*_3,205.4_ = 0.10, *P* = 0.959; month 3: *F*_3,205.2_ = 0.37, *P* = 0.774). However, in subsequent sampling months (4, 5, 6 and 7), there was a significant effect of dose on post-metamorphic body condition (LME: month 4: *F*_3,205.2_ = 5.63, *P* = 0.001; month 5: *F*_3,205.1_ = 4.53, *P* = 0.004; month 6: *F*_3,205.2_ = 7.61, *P* < 0.0001; month 7: *F*_3,205.3_ = 3.02, *P* = 0.031). Frogs supplemented with a dose of 1 or 10 mg g^−1^ exhibited lower body condition compared to frogs supplemented with 0 mg g^−1^ at months 4 and 5 (Tukey’s HSD test: month 4: *P* = 0.005 and *P* = 0.008 respectively; Fig. 3b), and lower body condition compared to doses of 0 mg g^−1^ (Tukey’s HSD test: *P* = 0.003 and *P* = 0.002 respectively) and 0.1 mg g^−1^ (Tukey’s HSD test: *P* = 0.011 and *P* = 0.008, respectively) at month 6. Frogs supplemented with 1 mg g^−1^ exhibited significantly higher body condition than those receiving 10 mg g^−1^ at month 7 (Tukey’s HSD test: *P* = 0.042).

Sex had a significant effect on body condition at month 0 (LME: *F*_3,207.6_ = 7.56, *P* = 0.007), whereby females were in better body condition than males. However, sex did not have a significant effect on body condition at months, 1, 2, 3, 4, 5, 6 and 7 (LME: month 1: *F*_1,208_ = 0.03, *P* = 0.852; month 2: *F*_1,207.3_ = 0.19, *P* = 0.660; month 3: *F*_1,207.9_ = 0.01, *P* = 0.934; month 4: *F*_1,207.7_ = 3.53, *P* = 0.062; month 5: *F*_1,207.7_ = 0.19, *P* = 0.661; month 6: *F*_1,207.6_ = 0.19, *P* = 0.667; month 7: *F*_1,207.9_ = 2.95, *P* = 0.087).

## Discussion

The influence of dietary carotenoids on vertebrate growth and development continues to be strongly debated because evidence for beneficial effects remains equivocal, both within and between taxa. One reason for this inconsistency might be that very few studies have tested for dose–response relationships or changes in effects across life stages. The aim of this study was to investigate the effect of varying doses of dietary beta-carotene supplements on the survival, growth and development of larval and adult *L. booroolongensis*. Our results show that there was no effect of supplement dose on the percentage of individuals surviving to metamorphosis, or to sexual maturity. However, there was a significant effect of supplement dose on larval growth and development, whereby individuals supplemented with an intermediate dose (1 mg g^−1^ beta-carotene) grew faster and metamorphosed significantly earlier than unsupplemented tadpoles. There was also an effect of dose on post-metamorphic growth, whereby individuals supplemented with a high dose (10 mg g^−1^ beta-carotene) exhibited slower growth (mass gain per month) than unsupplemented frogs 4–7 months post-metamorphosis. These findings provide evidence for a dose–response relationship and also indicate that the effects of beta-carotene supplementation differ between life stages.

Across all supplement doses, larval growth was exponential until tadpoles reached a body length of approximately 37 mm, at which point metamorphosis occurred. This developmental pattern indicates that *L. booroolongensis* has a minimum size threshold for metamorphosis. Supplementation with beta-carotene at an intermediate dose increased growth rate and enabled tadpoles to reach the size threshold for metamorphosis up to eight days earlier. This result provides support for our hypothesis that optimal doses of dietary carotenoids can assist vertebrate growth and development by scavenging ROS and promoting cellular function (Agarwal *et al.*, 2004). Of note, however, because we did not measure ROS levels, it is possible that beta-carotene improved growth via other mechanisms. For instance, beta-carotene is known to be a precursor to Vitamin A, which can have positive effects on bone development, immune function and visual-mediated foraging behaviour, all of which could improve growth rate (Ogilvy *et al.*, 2012). As such, the exact mechanism underpinning our finding that an intermediate beta-carotene dose expedites growth and development remains unknown. A critical next step will be to determine whether changes in dietary beta-carotene levels cause changes in ROS levels, indicative of altered antioxidant activity.

Our finding that larval growth and development was not improved at the low or high-supplement doses indicates that the effects of dietary beta-carotene were dose dependent. A lack of any effect at the low supplement dose may have resulted because beta-carotene was preferentially invested in other fitness-determining traits, such as immune function or exercise performance (as per the carotenoid trade-off hypothesis; Lin *et al.*, 2010). Alternatively, the concentration of beta-carotene used might simply have been too low to significantly alter cellular function and cause any significant change (Ogilvy *et al.*, 2012; Byrne and Silla, 2017). The lack of any beneficial effect at the high-supplement dose might have several explanations. First, above a certain threshold concentration beta-carotene might begin to have toxic effects, resulting in energy being diverted away from growth and into detoxifying and eliminating excess beta-carotene. Such toxic effects of carotenoids have been reported in other species (Palozza *et al.*, 1997; Cothran *et al.*, 2015). Alternatively, high concentrations might have elicited a mild pro-oxidant effect. Research with cell models has suggested that relatively high doses of beta-carotene can induce an overproduction of ROS due to either: (i) autoxidation of carotenoid metabolites, (ii) changes in the activity of cytochrome P450 enzymes and/or (iii) alterations to endogenous antioxidant defences (Palozza *et al.*, 2003). If high concentrations of beta-carotene have a similar pro-oxidant effect in Booroolong frogs, oxidative damage to cell structures could have slowed somatic growth, resulting in an overall reduction in developmental rate.

Dose-dependent effects were also evident post-metamorphosis. At the low supplement dose (0.1 mg g^−1^) there was no effect on post-metamorphic growth and development, but at both the intermediate (1 mg g^−1^) and high (10 mg g^−1^) doses there was evidence for negative growth effects. Significantly reduced body mass and body condition indicates that post-metamorphic frogs supplemented with intermediate to high doses of beta-carotene had reduced energy and protein reserves and were relatively unhealthy (Ferrie *et al.*, 2014). As for larvae, negative effects of high beta-carotene doses may have resulted from beta-carotene either having a toxic effect or causing pro-oxidant activity, with these effects being more pronounced in post-metamorphic animals. Irrespective of the exact cause, our finding that the effect of beta-carotene dose differed in larval versus post-metamorphic animals supports our second hypothesis that the effects of dietary beta-carotene supplementation are life stage-dependent. The most likely reason for this difference is that individuals produce much higher levels of ROS as larvae than as adults due to significantly faster rates of somatic growth prior to metamorphosis, making the protective antioxidant capacity of beta-carotene more important during early life. In support of this argument, the average percent weight gain of tadpoles per week was more than double that of post-metamorphic individuals. In principle, ROS production in tadpoles is also likely to have increased exponentially as larvae approached metamorphic climax because this is a developmental period that requires extremely high metabolic activity (Pough and Kamel, 1984).

Our findings have important conservation implications. Booroolong frogs are listed as critically endangered, and captive breeding and reintroduction are important recovery actions for this species. Supplying captive Booroolong frogs with dietary beta-carotene has the potential to support these actions in a number of ways. First, the capacity to more quickly generate large numbers of metamorphs might help managers to maintain colonies at optimal sizes and avoid issues associated with natural attrition and inbreeding. Second, more rapid generation of frogs could increase the number of individuals available for release, which could improve reintroduction success by overcoming issues linked to high dispersal, demographic stochasticity or low individual fitness at low population densities (i.e. the Allee effect; Armstrong and Seddon, 2008). Beyond benefits to growth, supplying frogs with dietary beta-carotene also has the potential to significantly improve reproductive success through positive effects on female fecundity and egg quality (see Ogilvy *et al.*, 2012; Dugas *et al.*, 2013). Such improvements to captive breeding protocols could reduce the costs of recovery programs and make them more financially viable, and sustainable (Tarszisz *et al.*, 2014). Given that anuran amphibians are declining faster than any other vertebrate group, and that captive breeding is a standard recovery action for threatened species globally, dietary supplementation of beta-carotene (and other carotenoids) could potentially benefit conservation programs for various threatened species. At present, however, it remains uncertain whether benefits to frogs are likely to be widespread. To date, the effect of carotenoids on amphibian growth and development has only been investigated in seven anuran species (including *L. booroolongensis*), and findings have been equivocal (Ogilvy and Preziosi, 2012; Ogilvy *et al.*, 2012; Dugas *et al.*, 2013; Cothran *et al.*, 2015; Byrne and Silla, 2017).

More broadly, our results add to the very small but growing body of evidence that dietary carotenoid supplements improve vertebrate growth and development. Carotenoids have been shown to influence growth and development in fish and birds, however, most studies have examined no more than two supplement doses, or have used a mixture of carotenoid compounds, or carotenoid derived substances, such as spirulina, shrimp head meal, and marigold petal meal (Boonyaratpalin and Unprasert, 1989; James *et al.*, 2006; Sinha and Asimi, 2007; Ezhil *et al.*, 2008; Güroy *et al.*, 2012; Arulvasu *et al.*, 2013). Without testing the effects of carotenoid compounds supplemented at three or more doses, it is impossible to attribute beneficial effects to specific carotenoids and identify optimal supplement doses. To the best of our knowledge, our study is the first to demonstrate dose-dependent and life stage-dependent effects of dietary beta-carotene supplementation on vertebrate growth and development. Considering the effect of carotenoids more broadly, only two studies (both in *Salmo salar*) have tested the effects of individual carotenoid compounds on growth and development at more than two doses. These studies both reported dose effects; fish fed higher doses of astaxanthin exhibited improved growth and survivorship (Christiansen *et al.*, 1995, Christiansen and Torrissen, 1996). Furthermore, these studies provided evidence for life-stage effects; juvenile fish required a higher dose of astaxanthin to exhibit enhanced growth and survivorship. In most vertebrates, growth rates will differ significantly between juvenile and adult life stages. As such, we predict that consideration of the effects of different doses of carotenoids across different life stages will reveal that dietary carotenoid supplementation has more widespread and profound effects on vertebrate growth and development than currently demonstrated.

## Supplementary Material

Supplementary Data

## References

[coy052C1] Agarwal A, Nallella KP, Allamaneni SSR, Said TM (2004) Review: role of antioxidants in treatment of male infertility: an overview of the literature. Reprod Biomed Online 8: 616–627. 15169573 10.1016/s1472-6483(10)61641-0

[coy052C2] Amar EC, Kiron V, Satoh S, Watanabe T (2004) Enhancement of innate immunity in rainbow trout (*Oncorhynchus mykiss walbaum*) associated with dietary intake of carotenoids from natural products. Fish Shellfish Immunol 16: 527–537. 15123294 10.1016/j.fsi.2003.09.004

[coy052C3] Armstrong DP, Seddon PJ (2008) Directions in reintroduction biology. Trends Ecol Evol 23: 20–25. 18160175 10.1016/j.tree.2007.10.003

[coy052C4] Arulvasu C, Meena SR, Chandhirasekar D, Sivaganam S (2013) Evaluation of natural sources of carotenoid pigments from *Rosa rubiginosa* on growth, survival and coloration of *Xiphophorus helleri* fish fry. Eur J Biol Res 5: 44–49.

[coy052C5] Blount JD, Houston DC, Møller AP (2000) Why egg yolk is yellow. Trends Ecol Evol 15: 47–49. 10652553 10.1016/s0169-5347(99)01774-7

[coy052C6] Boonyaratpalin M, Unprasert N (1989) Effects of pigments from different sources on colour changes and growth of red *Oreochromis niloticus*. Aquaculture 79: 375–380.

[coy052C7] Bouayed J, Bohn T (2010) Exogenous antioxidants—double-edged swords in cellular redox state: health beneficial effects at physiologic doses versus deleterious effects at high doses. Oxid Med Cell Longev 3: 228–237. 20972369 10.4161/oxim.3.4.12858 PMC2952083

[coy052C8] Byrne PG, Silla AJ (2017) Testing the effect of dietary carotenoids on larval survival, growth and development in the critically endangered southern corroboree frog. Zoo Biol 36: 161–169. 28198035 10.1002/zoo.21352

[coy052C9] Christiansen R, Glette J, Torrissen OJ, Waagbø R (1995) Antioxidant status and immunity in Atlantic salmon, *Salmo salar l*., fed semi‐purified diets with and without astaxanthin supplementation. J Fish Dis 18: 317–328.

[coy052C10] Christiansen R, Torrissen OJ (1996) Growth and survival of Atlantic salmon, *Salmo salar l.* Fed different dietary levels of astaxanthin. Juveniles. Aquacult Nutr 2: 55–62.

[coy052C11] Cothran RD, Gervasi SS, Murray C, French BJ, Bradley PW, Urbina J, Blaustein AR, Relyea RA (2015) Carotenoids and amphibians: effects on life history and susceptibility to the infectious pathogen, *Batrachochytrium dendrobatidis*. Conserv Physiol 3: cov005. 27293690 10.1093/conphys/cov005 PMC4778475

[coy052C12] Cucco M, Guasco B, Malacarne G, Ottonelli R (2006) Effects of β-carotene supplementation on chick growth, immune status and behaviour in the grey partridge, *Perdix perdix*. Behav Processes 73: 325–332. 16963199 10.1016/j.beproc.2006.08.002

[coy052C13] Deng J, Mai K, Ai Q, Zhang W, Wang X, Xu W, Liufu Z (2006) Effects of replacing fish meal with soy protein concentrate on feed intake and growth of juvenile Japanese flounder. Paralichthys olivaceus. Aquaculture 258: 503–513.

[coy052C14] Dias J, Huelvan C, Dinis MT, Métailler R (1998) Influence of dietary bulk agents (silica, cellulose and a natural zeolite) on protein digestibility, growth, feed intake and feed transit time in European seabass (*Dicentrarchus labrax*) juveniles. Aquat Living Resour 11: 219–226.

[coy052C15] Dugas MB, Yeager J, Richards-Zawacki CL (2013) Carotenoid supplementation enhances reproductive success in captive strawberry poison frogs (*Oophaga pumilio*). Zoo Biol 32: 655–658. 24151130 10.1002/zoo.21102

[coy052C16] Edge R, McGarvey D, Truscott T (1997) The carotenoids as anti-oxidants—a review. J Photochem Photobiol B 41: 189–200. 9447718 10.1016/s1011-1344(97)00092-4

[coy052C17] Ezhil J, Jeyanthi C, Narayanan M (2008) Marigold as a carotenoid source on pigmentation and growth of red swordtail, *Xiphophorus helleri*. *Turk*. J Fish Aquat Sci 8: 99–101.

[coy052C18] Ferrie GM, Alford VC, Atkinson J, Baitchman E, Barber D, Blaner WS, Crawshaw G, Daneault A, Dierenfeld E, Finke M (2014) Nutrition and health in amphibian husbandry. Zoo Biol 33: 485–501. 25296396 10.1002/zoo.21180 PMC4685711

[coy052C19] Gascon C, Collins JP, Moore RD, Church DR, Mckay JE, Mendelson Iii JR (2007) Amphibain conservation action plan. UK IUCN/SSC Amphibian Specialist group, Gland, Switzerland and Cambridge.

[coy052C20] Gillooly JF, Charnov EL, West GB, Savage VM, Brown JH (2002) Effects of size and temperature on developmental time. Nature 417: 70. 11986667 10.1038/417070a

[coy052C21] Güroy B, Şahin İ, Mantoğlu S, Kayalı S (2012) Spirulina as a natural carotenoid source on growth, pigmentation and reproductive performance of yellow tail cichlid Pseudotrop*heus acei*. Aquac Int 20: 869–878.

[coy052C22] Hero JM, Gillespie G, Lemckert F, Robertson P, Littlejohn M (2004) *Litoria booroolongensis. The IUCN Red List of Threatened Species 2004*: e.T41029A10390615.

[coy052C23] James R, Sampath K, Thangarathinam R, Vasudevan I (2006) Effect of dietary spirulina level on growth, fertility, coloration and leucocyte count in red swordtail, *Xiphophorus helleri*. Isr J Aquac 58: 97–104.

[coy052C24] Keogh LM, Byrne PG, Silla AJ (2018) Effect of long-term dietary beta-carotene supplementation on sperm concentration and motility in an endangered amphibian. Anim Reprod Sci 195: 259–265. 10.1016/j.anireprosci.2018.06.003 31262404

[coy052C25] Lin SM, Nieves-Puigdoller K, Brown AC, McGraw KJ, Clotfelter ED (2010) Testing the carotenoid trade-off hypothesis in the polychromatic midas cichlid, *Amphilophus citrinellus*. Physiol Biochem Zool 83: 333–342. 20151818 10.1086/649965

[coy052C26] Marri V, Richner H (2014) Yolk carotenoids increase fledging success in great tit nestlings. Oecologia 176: 371–377. 25142046 10.1007/s00442-014-3051-2

[coy052C27] Ogilvy V, Preziosi RF (2012) Can carotenoids mediate the potentially harmful effects of ultraviolet light in *Silurana (Xenopus) tropicalis* larvae? J Anim Physiol Anim Nutr (Berl) 96: 693–699. 21793941 10.1111/j.1439-0396.2011.01197.x

[coy052C28] Ogilvy V, Preziosi RF, Fidgett AL (2012) A brighter future for frogs? The influence of carotenoids on the health, development and reproductive success of the red-eye tree frog. Anim Conserv 15: 480–488.

[coy052C29] Palozza P, Luberto C, Calviello G, Ricci P, Bartoli GM (1997) Antioxidant and prooxidant role of β-carotene in murine normal and tumor thymocytes: effects of oxygen partial pressure. Free Radic Biol Med 22: 1065–1073. 9034246 10.1016/s0891-5849(96)00498-4

[coy052C30] Palozza P, Serini S, Di Nicuolo F, Piccioni E, Calviello G (2003) Prooxidant effects of β-carotene in cultured cells. Mol Aspects Med 24: 353–362. 14585306 10.1016/s0098-2997(03)00031-1

[coy052C31] Pough FH, Kamel S (1984) Post-metamorphic change in activity metabolism of anurans in relation to life history. Oecologia 65: 138–144. 28312123 10.1007/BF00384476

[coy052C32] Řehulka J (2000) Influence of astaxanthin on growth rate, condition, and some blood indices of rainbow trout. Oncorhynchus mykiss. Aquaculture 190: 27–47.

[coy052C33] Silla AJ, McInerney EP, Byrne PG (2016) Dietary carotenoid supplementation improves the escape performance of the southern corroboree frog. Anim Behav 112: 213–220.

[coy052C34] Sinha A, Asimi OA (2007) China rose (*Hibiscus rosasinensis*) petals: a potent natural carotenoid source for goldfish (*Carassius auratus l.*). Aquac Res 38: 1123–1128.

[coy052C35] Stoss J, Refstie T (1983) Short-term storage and cryopreservation of milt from Atlantic salmon and sea trout. Aquaculture 30: 229–236.

[coy052C36] Takaichi S (2011) Carotenoids in algae: distributions, biosyntheses and functions. Mar Drugs 9: 1101–1118. 21747749 10.3390/md9061101 PMC3131562

[coy052C37] Tarszisz E, Dickman CR, Munn AJ (2014) Physiology in conservation translocations. Conserv Physiol 2: cou054. 27293675 10.1093/conphys/cou054 PMC4732500

[coy052C38] Tella JL, Figuerola J, Negro JJ, Blanco G, Rodríguez‐Estrella R, Forero MG, Blazquez MC, Green AJ, Hiraldo F (2004) Ecological, morphological and phylogenetic correlates of interspecific variation in plasma carotenoid concentration in birds. J Evol Biol 17: 156–164. 15000658 10.1046/j.1420-9101.2003.00634.x

[coy052C39] Toomey MB, McGraw KJ (2011) The effects of dietary carotenoid supplementation and retinal carotenoid accumulation on vision-mediated foraging in the house finch. PLoS One 6: e21653. 21747917 10.1371/journal.pone.0021653 PMC3126843

[coy052C40] Torrissen OJ (1984) Pigmentation of salmonids—effect of carotenoids in eggs and start-feeding diet on survival and growth rate. Aquaculture 43: 185–193.

[coy052C41] Wang Y-J, Chien Y-H, Pan C-H (2006) Effects of dietary supplementation of carotenoids on survival, growth, pigmentation, and antioxidant capacity of characins, Hyphessobrycon *callistus*. Aquaculture 261: 641–648.

[coy052C42] Wilbur HM, Collins JP (1973) Ecological aspects of amphibian metamorphosis. Science 182: 1305–1314. 17733097 10.1126/science.182.4119.1305

