# Supplementary material for: Dose and life stage-dependent effects of dietary beta-carotene supplementation on the growth and development of the Booroolong frog
Source: Conserv Physiol. 2018 Sep 19;6(1):coy052. doi: 10.1093/conphys/coy052 (PMC6144775; doi:10.1093/conphys/coy052)
Supplement: Supplementary Data [file coy052_supporting_information.doc]

**Supplementary Material**

**Dose and life-stage dependent effects of dietary beta-carotene supplementation on the growth and development of the Booroolong frog**

*Leesa M. Keogh^1*^, Aimee J. Silla^1^, Michael McFadden^2^, and Phillip G. Byrne^1^*

*^1^* Centre for Sustainable Ecosystem Solutions,
School of Earth, Atmospheric and Lifesciences
University of Wollongong, NSW, Australia 2522

^2^ Herpetofauna Department,

Taronga Conservation Society Australia,

PO Box 20, Mosman, NSW, Australia 2088

*** Corresponding author email: lk549@uowmail.edu.au

**Supplementary Fig. S1** Schematic of the layout of the experimental room; each row represents a shelf in temperature control room (1-4). Shelf one consisted of the pilot study experimental animals and shelves 2, 3 & 4 consisted of animals in the final dietary experiment. Each box represents an individual tadpole/frog and letters A through D represent each clutch and their allocation across the experimental room. Trays 1-41 represent the plastic trays, which housed 9 individual aquaria.

**Supplementary Table S2** Composition of experimental diets supplied during the larval life stage.

| Treatment | Fish flake (mg) | Beta-carotene (mg) | Cellulose (mg) | Overall mass of food (mg) |
| --- | --- | --- | --- | --- |
| 0 mg g^-1^ | 990 | 0.0 | 10.0 | 1000 |
| 0.1 mg g^-1^ | 990 | 0.1 | 9.9 | 1000 |
| 1 mg g^-1^ | 990 | 1.0 | 9.0 | 1000 |
| 10 mg g^-1^ | 990 | 10.0 | 0.0 | 1000 |

**Supplementary Table S2** Carotenoid composition of the basal diet fed to larval and post-metamorphic *L.booroolongensis*

|  | Larval diet | | Post-metamorphic diet | |
| --- | --- | --- | --- | --- |
| Carotenoid compound | %TC | mg g^-1^ | %TC | mg g^-1^ |
| Astaxanthin | 1.60 | 0.000233 | 1.45 | 0.000076 |
| Canary xanthophyll B | 1.40 | 0.000204 | 3.02 | 0.000159 |
| α-Doradexanthin | 1.60 | 0.000233 | 0.02 | 0.000001 |
| Canary xanthophyll A | 5.10 | 0.000741 | 20.57 | 0.001081 |
| Lutein | 15.60 | 0.002268 | 16.67 | 0.000876 |
| Zeaxanthin | 6.30 | 0.000916 | 0.53 | 0.000028 |
| 3’-Dehydrolutein | 2.70 | 0.000392 | 1.58 | 0.000083 |
| Adonirubin | 5.90 | 0.000858 | 0.72 | 0.000038 |
| Echinenone | 0.00 | <0.000001 | 0.00 | <0.000001 |
| Canthaxanthin | 1.10 | 0.000160 | 0.65 | 0.000034 |
| Anhydrolutein 1 | 0.30 | 0.000044 | 0.03 | 0.000002 |
| Anhydrolutein 2 | 0.00 | <0.000001 | 0.13 | 0.000007 |
| Anhydrolutein 3 | 1.30 | 0.000189 | 1.80 | 0.000095 |
| β-Cryptoxanthin | 12.20 | 0.001773 | 13.37 | 0.000702 |
| Lycopene 1 | 0.00 | <0.000001 | 5.70 | 0.000300 |
| Lycopene 2 | 5.30 | 0.000770 | 2.02 | 0.000106 |
| Lycopene 3 | 0.00 | <0.000001 | 0.00 | <0.000001 |
| α-Carotene | 0.00 | <0.000001 | 0.00 | <0.000001 |
| β-Carotene | 37.60 | 0.005466 | 20.30 | 0.001067 |
| Unknown 1 | 2.00 | 0.000291 | 11.45 | 0.000602 |
| Total carotenoids | 100.00 | 0.014536 | 100.00 | 0.005255 |

Results of reverse-phase high-performance liquid chromatography conducted by the Animal Health and Research Centre at Adelaide Zoo. %TC = percentage of total carotenoids

**Complimentary experiment: the effect of cellulose on larval growth and development in the Booroolong Frog**

**5.1 Methods**

A complementary experiment was conducted in parallel to our main study and aimed to determine whether the cellulose additive had an affect on larval growth and development. Tadpoles were reared to metamorphosis under one of two dietary treatments, with 36 replicate individuals per treatment. Treatment 1 consisted of tadpoles reared on a basal only diet, which consisted of 990 mg ground fish flake mixture (75:25 mixture of Sera Flora/ Sera Sans; SERA, Germany) and suspended in 10 mL of reverse-osmosis water. Treatment 2 consisted of tadpoles reared on a cellulose-supplemented diet, which consisted of 990 mg ground fish flake mixture, 10 mg of cellulose (435236; Sigma-Aldrich, Castle Hill, NSW) and suspended in 10 mL of reverse-osmosis water. Animals were individually housed in plastic containers (10 cm diameter and 10.5 cm high) and were held in groups of nine containers per plastic drainage tray, with each row as alternating treatments across one shelf in the experimental room (Fig. S1). The room was artificially illuminated on a 15 :9 hour, day: night cycle (including twilight lighting for half an hour each cycle to simulate dawn and dusk). In addition to overhead room lighting, UV-B lights (Reptisum 10.0 UVB 3600 bulb; Pet Pacific, Australia) were suspended approximately 20 cm above the shelf, providing 6.5 hours per day of UV-B light (between 10 am to 4:30 pm). Ambient temperature in the room was maintained at 22 °C (range was 21.9 °C to 23 °C).

**5.2 Results**

**Supplementary Table S3** Complementary experiment: effect of cellulose supplementation (0 & 10 mg g^-1^) on time to metamorphosis, mass at metamorphosis and survivorship (percentage) in Booroolong frogs (n=72). Results from one-way ANOVAs are presented for time to metamorphosis data and mass at metamorphosis data and results from Chi-squared likelihood ratio test for survivorship data. Data present are untransformed means ± SEM. Experimental treatments connected by the same letter are not significantly different from one another (P>0.05).

|  | Treatment | | F_d.f_.  Or x^2^ | *P* value |
| --- | --- | --- | --- | --- |
|  | Cellulose | Basal |  |  |
| Time to metamorphosis (days) | 80.34^a^ ± 1.20 | 77.85^a^ ± 1.15 | 2.61_1,61_ | 0.1111 |
| Mass at metamorphosis (g) | 0.28^a^ ± 0.006 | 0.26^a^ ± 0.005 | 2.27_1,61_ | 0.1368 |
| Survivorship (%) | 88.9 | 91.7 | 0.159_1_ | 0.6903 |
